# Coccidioidomycosis in New York State.

**DOI:** 10.3201/eid0601.000104

**Published:** 2000

**Authors:** V Chaturvedi, R Ramani, S Gromadzki, B Rodeghier, H G Chang, D L Morse

**Affiliations:** New York State Department of Health, Albany, New York 12208-2002, USA. vishnu@wadsworth.org

## Abstract

Coccidioidomycosis, a systemic fungal disease caused by Coccidioides immitis, is endemic in the southwestern United States and in parts of Mexico and Central and South America. Only sporadic cases have been reported in areas (including New York) where the disease is not endemic. We used hospital discharge records and state mycology laboratory data to investigate the characteristics of C. immitis infections among New York State residents. From 1992 to 1997, 161 persons had hospital discharge diagnoses of coccidioidomycosis (ICD9 Code 114.0 - 114.5, 114.9). From 1989 to 1997, 49 cultures from patients were confirmed as C. immitis; 26 of these patients had traveled to disease-endemic areas. Fourteen of 16 isolates had multilocus genotypes similar to those of Arizona isolates, which corroborates the travel-related acquisition of the disease. Our results indicate that coccidioidomycosis may be more common in New York residents than previously recognized. Increased awareness among health-care providers should improve timely diagnosis of coccidioidomycosis and prevention of associated illnesses and deaths among patients in nondisease-endemic areas.


### Coccidioidomycosis in New York State

**Vishnu Chaturvedi,* Rama Ramani,* Sally Gromadzki,* Birgit Rodeghier,* Hwa-Gan Chang,† and Dale L. Morse*†**
New York State Department of Health, Albany, New York, USA; and †School of Public Health, University at Albany, SUNY, Albany, New York, USA

Coccidioidomycosis, a systemic fungal disease caused by *Coccidioides immitis,* is endemic in the southwestern United States and in parts of Mexico and Central and South America. Only sporadic cases have been reported in areas (including New York) where the disease is not endemic. We used hospital discharge records and state mycology laboratory data to investigate the characteristics of *C. immitis* infections among New York State residents. From 1992 to 1997, 161 persons had hospital discharge diagnoses of coccidioidomycosis (ICD9 Code 114.0 - 114.5, 114.9). From 1989 to 1997, 49 cultures from patients were confirmed as *C. immitis*; 26 of these patients had traveled to disease-endemic areas. Fourteen of 16 isolates had multilocus genotypes similar to those of Arizona isolates, which corroborates the travel-related acquisition of the disease. Our results indicate that coccidioidomycosis may be more common in New York residents than previously recognized. Increased awareness among health-care providers should improve timely diagnosis of coccidioidomycosis and prevention of associated illnesses and deaths among patients in nondisease-endemic areas.

Coccidioidomycosis, a systemic disease caused by the dimorphic fungus *Coccidioides immitis,* is endemic in the southwestern United States and parts of Mexico and in Central and South America (1,2). The incidence of this systemic mycosis in disease-endemic areas increased during the 1990s (3-5). An estimated 100,000 infections occur in the United States annually, and 1 in 200 infections progresses to disseminated disease (1,6). Sporadic cases of coccidioidomycosis have been reported among visitors to the Southwest, and one earlier report recognized coccidioidomycosis as a serious travel hazard for visitors to that region (7-9).

*C. immitis,* a soil fungus, inhabits a unique ecologic niche in the topsoil of the lower Sonoran life zone (10). The infectious propagules are arthroconidia, single-cell fragments of mycelial threads, which become easily airborne to cause inhalation exposure. In the alveoli, arthroconidia undergo dimorphic transition to spherules, which fragment into endospores. When released from the spherule, each endospore can act as a new infectious unit in vivo (1). *C. immitis,* one of the most virulent and infectious fungal pathogens, poses a serious occupational hazard for laboratory personnel, especially in areas where the disease is not endemic and workers are less likely to practice biohazard safety level (BSL)-3 containment, which is required for the handling of this pathogen. The serious biohazard potential of *C. immitis* has led to its inclusion among the biological agents covered under the recently enacted Anti-Terrorist and Effective Death Penalty Act, which regulates interstate transport of infectious materials (11).

The extent and source of *C. immitis* infections have not been thoroughly investigated in areas where the disease is not endemic. In this study we summarize discharge data from persons with coccidioidomycosis hospitalized in New York, a nondisease-endemic area with a single coccidioidomycosis case report (7). In addition, we tested a proposed set of molecular markers for multilocus genotyping of *C. immitis* to determine the geographic origins of fungal isolates obtained in this study.

Address for correspondence: Vishnu Chaturvedi, Mycology Laboratory, Wadsworth Center, New York State Department of Health, 120 New Scotland Ave., Albany, NY 12208-2002, USA; fax 518-486-7971; e-mail: vishnu@wadsworth.org.

### The Study

Data on hospitalization for coccidioidomycosis in the state of New York from 1992 to 1997 were obtained from the New York Statewide Planning and Research Cooperative System, which compiles uniform inpatient data from all general acute-care hospitals (all public and private hospitals and hospital-based and free-standing ambulatory surgery facilities).

Patients were selected if their hospitalizations listed a primary or secondary discharge diagnosis of coccidioidomycosis (ICD9 114.0-114.5, 114.9) during 1992 to 1997. Information was collected from hospital discharge data abstracts and uniform billing forms for age at admission, sex, race, ethnicity, county of residence, insurance status, seasonality, disposition of patient, hospital length of stay, and total hospital cost.

Hospital medical records were requested from the 16 patients with positive *C. immitis* cultures available for multilocus genotyping (1994-1997). These records were reviewed for travel histories.

### Laboratory Methods

All suspect *C. immitis* isolates were transported and processed with full safety precautions in a BSL-3 facility. The isolates were subcultured on modified Sabouraud's agar and Mycosel agar, morphologic and microscopic features were examined, and cultures were processed for specific nucleic acid detection. The AccuPROBE *C. immitis* culture identification test (Gen-Probe Inc., San Diego, CA) was used to confirm the fungal identity. Before 1993, most culture identifications were confirmed by using a *C. immitis* exoantigen test kit (Scott Laboratories Inc., Fiskeville, RI).

The fungal DNA was extracted from mycelial cultures according to the method of Pan et al. (12). PCR-RFLP was done as described by Burt et al. (13), with minor modifications. We studied five of the 10 loci originally used for each *C. immitis* isolate because analysis showed these loci to be most useful for strain differentiation (13). The loci/restriction enzymes used included a1/*BsrI*, bg/*DdeI*, bl/*DdeI*, bq/*HinFI,* and e1/*BsmI*. Accordingly, California isolates were expected to show polymorphism at e1, and Arizona isolates could be either positive or negative at each locus, while isolates from Texas would be polymorphic only for the a1, bq, and e1 loci.

### Findings

Hospital discharge data from 1992 to 1997 in New York showed 181 hospitalizations with coccidioidomycosis as the primary or secondary discharge diagnoses (Table 1). The yearly distribution of hospitalizations was 30 (1992), 28 (1993), 30 (1994), 33 (1995), and 32 (1996), with no discernible seasonal pattern. Coccidioidomycosis was the primary diagnosis in 75 hospitalizations and the secondary diagnosis in 106. The clinical diagnoses according to ICD9 Codes included 105 hospitalizations for pulmonary coccidioidomycosis (114.0, 114.5), one with extrapulmonary coccidioidomycosis (114.1), five with coccidioidal meningitis (114.2), 18 with other forms of coocidioidomycosis (114.3), 19 with chronic pulmonary coccidioidomycosis (114.4), and 33 with unspecified coccidioidomycosis (114.9). The mean length of stay was 16 days, with an average hospital cost of $22,516.00.

One hundred sixty-one patients were hospitalized, of whom five had two hospitalizations, two had three, one had four, and one had nine (Table 1). Thirty-two (20%) patients had HIV, 16 patients (10%) had cancer, and 18 patients (11%) died while hospitalized. Overall,

Table 1. Coccidioidomycosis hospital discharge data, New York State, 1992–1997

| Characteristic | N | (%) |
|---|---|---|
| Total hospitalizations | 181 | |
| Total patients | 161 | |
| Male | 93 | (58) |
| Female | 68 | (42) |
| Race | | |
| White | 128 | (79) |
| Black | 18 | (11) |
| Asian/Pacific Islander | 2 | ( 1) |
| Other | 9 | ( 6) |
| Unknown | 4 | ( 3) |
| Ethnicity | | |
| Hispanic | 4 | ( 2) |
| Non-hispanic | 151 | (94) |
| Unknown | 6 | ( 4) |
| Coccidioidomycosis diagnosis | | |
| Primary | 75 | (41) |
| Secondary | 106 | (59) |
| Concurrent diagnosis | | |
| HIV | 32 | (20) |
| Cancer | 16 | (10) |
| Died while in hospital | 18 | (11) |
| Mean length of stay (days) | 16 | |
| Age range (yrs) | 0-93 (median 58) | |
| Mean hospital charge | $22,516.00 | |

120 (75%) of these 161 patients were either >54 years of age, had cancer, or were HIV infected.

Mean and median ages for HIV-infected patients were 36 and 33 years, compared with 66 and 64 years for cancer patients and 59 and 63 years for others. Further comparison of HIV-infected patients with patients who had neither cancer nor HIV infection showed differences in percentages with a primary diagnosis of coccidioidomycosis (22% vs 50%), male sex (81% vs 50%), black race (31% vs 5%), and death during hospitalization (31% vs 6%).

Forty-nine *C. immitis* cultures were identified at the state mycology laboratory from 1989 to 1997 (Figure).

Sixteen patient isolates were available for multilocus genotyping. The hospital records of these patients were reviewed to determine the likely source of infection. All had a history of travel to the Southwest, with 12 of 16 patients traveling to Arizona. However, date(s) of travel could not be correlated with onset of the disease. The typing of five alleles allowed unambiguous matches with Arizona genotypes for eight isolates from patients whose histories supported this conclusion (Table 2). It was also possible to type the respective *C. immitis* isolates to Arizona in five instances in which patients gave a history of travel to more than one disease-endemic area (four with travel to Arizona). The Arizona genotypes of three isolates (474-97, 639-97, and 779-97) did not match, as these patients had a history of travel to California, California and Mexico, and California, respectively.

### Conclusions

Before 1994, the Centers for Disease Control and Prevention (CDC) definition for coccidioidomycosis relied solely on a physician's clinical diagnosis (5). The current Council of State and Territorial Epidemiologists/CDC surveillance case definition requires the presence of both clinically compatible symptoms and laboratory evidence of infection (5). The laboratory criteria include a positive culture, positive histopathologic results, molecular evidence of *C. immitis,* a positive serologic test, or a positive skin test. The retrospective nature of this study and our

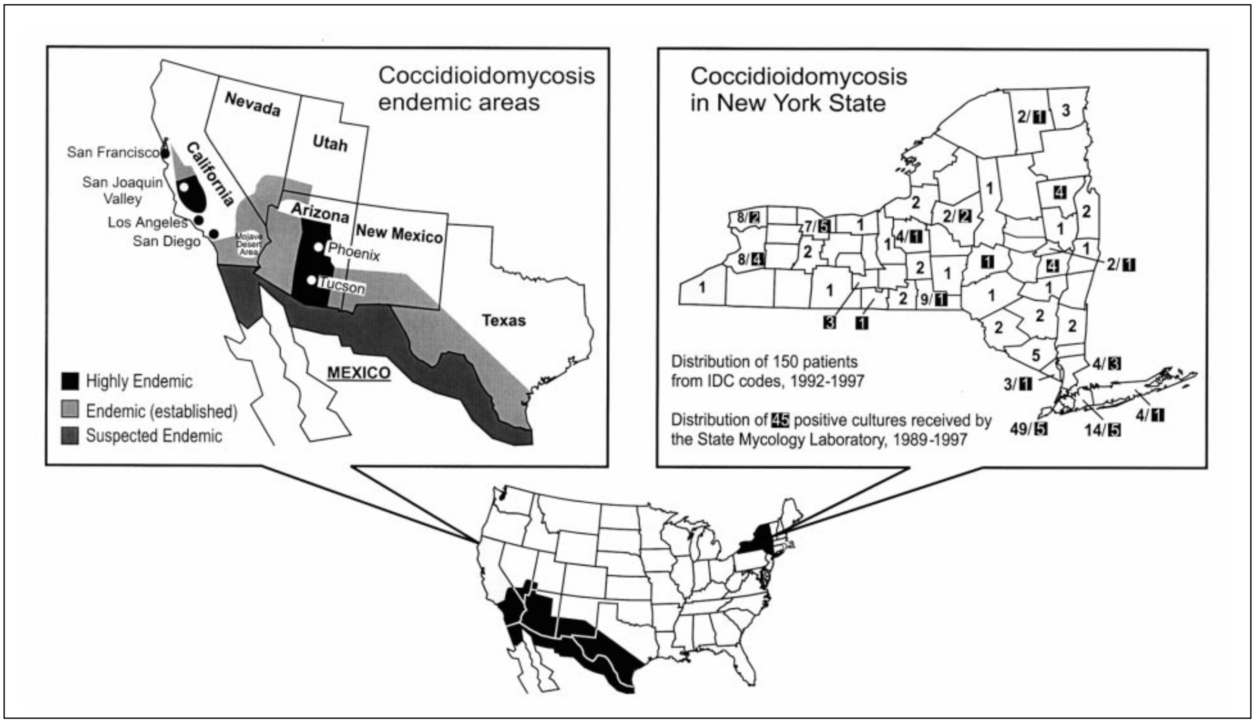

Figure. Coccidioidomycosis in New York, 1989–1997. The figure on the left highlights coccidioidomycosis-endemic areas in the United States (adapted from Kirkland TN, Fierer [2]). The figure on the right depicts New York countywide distribution of 150 out of 161 patients in the discharge records (1992–1997); the highlighted numbers show counties from which 45 of the 49 *Coccidioides immitis* cultures were referred to the state mycology laboratory (1989–1997).

Table 2. Correlation among clinical history, travel, and multilocus genotypes of *Coccidioides immitis* isolates from 16 patients, New York, 1994–1997

| Isolate no. | Clinical diagnosis | Treatment[a] outcome | Travel history[b] | Multilocus[c] genotype | Typing pattern |
|---|---|---|---|---|---|
| 104-94 | - | - | AZ | 01101 | AZ |
| 376-95 | Pneumonia | Itra/discharged | AZ | 11101 | AZ |
| 515-95 | Immunocomp.[d] | Amp B, Itra/died | AZ/CA | 11100 | AZ |
| 750-95 | - | - | AZ | 11101 | AZ |
| 131-96 | Pneumonia | Flu/discharged | AZ/TX | 11100 | AZ |
| 229-96 | Immunocomp. | Amp B, Flu, Itra/died | AZ | 11101 | AZ |
| 366-96 | Pneumonia | No treatment/discharged | AZ | 11100 | AZ |
| 819-96 | Immunocomp. | Amp B, Itra/died | AZ/CA | 11100 | AZ |
| 269-97 | Lung mass | No treatment/discharged | AZ | 11100 | AZ |
| 371-97 | Immunocomp. | Amp B,Flu,Itra/discharged | AZ | 11100 | AZ |
| 474-97 | Hydrocephalus | Flu/discharged | CA | 11101 | AZ |
| 588-97 | Pneumonia | Amp B,Flu/discharged | AZ | 11101 | AZ |
| 639-97 | Lung mass | Thoracotomy/discharged | CA/Mexico | 11101 | AZ |
| 686-97 | - | - | Southwest | 11100 | AZ |
| 779-97 | Pneumonia | Itra/discharged | CA | 11100 | AZ |
| 815-97 | URI[e] | Flu/discharged | AZ/CA | 11101 | AZ |

[a]Amp B, Amphotericin B; Flu, Fluconazole; Itra, Itraconazole.
[b]AZ, Arizona; CA, California; TX, Texas.
[c]The presence of polymorphism was scored as 1 and its absence as 0; every isolate was assigned a five-numeral genotype.
[d]Immunocomp, immunocompromised.
[e]URI, upper respiratory infection.

exclusive reliance on hospital discharge summary data precluded detailed evaluation of diagnostic criteria. Nevertheless, the average of 30 hospital discharges per year suggests that coccidioidomycosis may be more common in New York residents than previously recognized. The largest number of positive cases was reported from New York City, but the rate was not increased, and cases were reported throughout the state. Furthermore, while information on travel history was limited, all 16 patients from whom information was obtained had traveled to disease-endemic areas before becoming ill.

Immunocompromised persons, infants, and non-Caucasians are considered at increased risk for coccidioidomycosis (1). The mean age of patients in this series was 54 years, which is in agreement with >40-year age-group most commonly diagnosed in disease-endemic areas (5,6). As expected, a large proportion of patients had other diagnoses consistent with underlying immunosuppression, including a primary diagnosis of HIV in 20% and cancer in 10%. Overall, 75% were  55 years of age, had cancer, or were HIV infected. The availability of *C. immitis* cultures from only 10% of cases precluded any definitive investigations of geographic areas in which New Yorkers are at greater risk for infection.

The discovery of recombination in clinical isolates of *C. immitis* (14) has shown genetic evidence of two sexually distinct taxa in this pathogen, which was previously thought to reproduce asexually (15). A corollary of these investigations was the development of a multilocus genotyping scheme for determining the geographic origin of clinical isolates. Although the discharge data available for this study did not include travel information, we were able to delineate Arizona isolates in a subpopulation of our study for which cultures and medical record travel data were available. Thus, a patient's travel history was correlated with disease acquisition in a particular area by independently demonstrating the geographic pattern of the patient's isolate in 13 (81%) of 16 patients evaluated. However, the typing scheme could not determine the geographic origin of two *C. immitis* isolates, which came from patients thought to have traveled to both California and Mexico. This discrepancy may have resulted from the limited number of alleles used for typing, incomplete travel histories, shortcomings of the typing scheme for California isolates, or the possibility that the two isolates belonged to an outlier group of strains. Further testing is needed on a larger set of isolates from different geographic areas to evaluate this typing scheme.

As a result of this study, laboratories participating in the New York State Clinical Laboratory Evaluation Program (Mycology) were alerted about the hazards posed by *C. immitis*. A safe procedure for initial examination of suspect cultures was recommended. The laboratories were also provided with guidelines for safe transport of *C. immitis* to the state mycology laboratory for confirmation and characterization.

The diagnosis and clinical management of coccidioidomycosis in areas where the disease is not endemic pose unique challenges. The clinical symptoms of the disease mimic those of other infectious diseases, which may result in misdiagnosis and inappropriate treatment (1,16). Additionally, *C. immitis* cultures could be easily confused with a number of nonpathogenic fungi by unsuspecting laboratory personnel, who are at risk for infection. Increased awareness among health-care providers is likely to help in the timely diagnosis of coccidioidomycosis and the prevention of associated illness and death among patients in nondisease-endemic areas.

### Acknowledgments

This study was partially supported by Centers for Control and Prevention Emerging Pathogens Program funds (DLM) and an unrestricted educational grant from Pfizer, Inc. (VC), and was presented as a poster at the First International Conference on Emerging Infectious Diseases, Atlanta, Georgia, March 8-11, 1998.

Dr. Chaturvedi is director of the Mycology Laboratory, Wadsworth Center, New York State Department of Health, and assistant professor of Biomedical Sciences, School of Public Health, SUNY, Albany. His research interests are molecular epidemiology, fungal pathogenesis, and antifungal drug discovery.

### References

1. Stevens DA. Current concepts: coccidioidomycosis. N Engl J Med 1995;332:1077-82.
2. Kirkland TM, Fierer J. Coccidioidomycosis: a reemerging infectious disease. Emerg Infect Dis 1996;2:192-9.
3. Centers for Disease Control and Prevention. Coccidioidomycosis—United States, 1991-1992. MMWR Morb Mortal Wkly Rep 1993;42:21-4.
4. Pappagianis D. Marked increase in cases of coccidiodomycosis in California: 1991, 1992, and 1993. Clin Infect Dis 1994;19 Suppl 1:S14-8.
5. Centers for Disease Control and Prevention. Coccidioidomycosis—Arizona, 1990-1995. MMWR Morb Mortal Wkly Rep 1996;49:1069-73.
6. Schneider E, Hajjeh RA, Spiegel RA, Jibson RW, Harp EL, Marshall Ga, et al. Coccidioidomycosis outbreak following the Northridge, California, earthquake. JAMA 1997;277:904-8.
7. Smith MA, Anderson AE, Kostroff K. An unusual case of coccidioidomycosis. J Clin Microbiol 1994;32:1063-4.
8. Ogiso A, Ito M, Koyama M, Yamaoka H, Hotchi M, McGinnis MR. Pulmonary coccidioidomycosis in Japan: case report and review. Clin Infect Dis 1997;25:1260-1.
9. Harrell ER, Honeycut WM. Coccidiodomycosis: a traveling fungus disease. Arch Dermatol 1963;87:98-106.
10. Fiese MJ. Coccidioidomycosis. Springfield (IL): Charles C. Thomas Publ.; 1958. p. 79-80.
11. Antiterrorism and Effective Death Penalty Act of 1996, Pub. L. No. 104-132, 110 Stat. 1214 (codified as amended in scattered sections of the U.S.C.).
12. Pan S, Sigler L, Cole GT. Evidence for a phylogenetic connection between *Coccidioides immitis* and *Uncinocarpus reesii* (Onygenaceae). Microbiology 1994;140:1481-94.
13. Burt A, Dechairo BM, Koening GL, Carter DA, White TJ, Taylor JW. Molecular markers reveal differentiation among isolates of *Coccidioides immitis* from California, Arizona and Texas. Mol Ecol 1997;6:781-6.
14. Burt A, Carter DA, Koenig GL, White TJ, Taylor JW. Molecular markers reveal cryptic sex in the human pathogen *Coccidioides immitis*. Proc Natl Acad Sci U S A 1996;93:770-3.
15. Koufopanou V, Burt A, Taylor JW. Concordance of gene genealogies reveals reproductive isolation in the pathogenic fungus *Coccidioides immitis*. Proc Natl Acad Sci U S A 1997;94:5478-82.
16. Rippon JW. Medical mycology: the pathogenic fungi and the pathogenic actinomycetes. 3rd ed. Philadelphia (PA): W.B. Saunders Co.; 1988.
